# Shugoshin 1 is dislocated by KSHV-encoded LANA inducing aneuploidy

**DOI:** 10.1371/journal.ppat.1007253

**Published:** 2018-09-13

**Authors:** Fengchao Lang, Zhiguo Sun, Yonggang Pei, Rajnish Kumar Singh, Hem Chandra Jha, Erle S. Robertson

**Affiliations:** Departments of Otorhinolaryngology–Head and Neck Surgery, and Microbiology, the Tumor Virology Program, Abramson Cancer Center, Perelman School of Medicine at the University of Pennsylvania, Philadelphia, PA, United States of America; Northwestern University, UNITED STATES

## Abstract

Shugoshin-1 (Sgo1) protects the integrity of the centromeres, and H2A phosphorylation is critical for this process. The mitotic checkpoint kinase Bub1, phosphorylates H2A and ensures fidelity of chromosome segregation and chromosome number. Oncogenic KSHV induces genetic alterations through chromosomal instability (CIN), and its essential antigen LANA regulates Bub1. We show that LANA inhibits Bub1 phosphorylation of H2A and Cdc20, important for chromosome segregation and mitotic signaling. Inhibition of H2A phosphorylation at residue T120 by LANA resulted in dislocation of Sgo1, and cohesin from the centromeres. Arrest of Cdc20 phosphorylation also rescued degradation of Securin and Cyclin B1 at mitotic exit, and interaction of H2A, and Cdc20 with Bub1 was inhibited by LANA. The N-terminal nuclear localization sequence domain of LANA was essential for LANA and Bub1 interaction, reversed LANA inhibited phosphorylation of H2A and Cdc20, and attenuated LANA-induced aneuploidy and cell proliferation. This molecular mechanism whereby KSHV-induced CIN, demonstrated that the NNLS of LANA is a promising target for development of anti-viral therapies targeting KSHV associated cancers.

## Author summary

KSHV is a known oncogenic herpes virus associated with human malignancies and lymphoproliferative disorders, which includes Kaposi’s sarcoma, Primary effusion lymphoma, and Multicentric Castleman’s disease. KSHV disrupts the G1 and G2/M checkpoints through multiple pathways. Whether KSHV can directly interfere with spindle checkpoints is not known. Impairment of the mitotic checkpoint protein Bub1 leads to CIN and oncogenesis through displacement of Shugoshin-1. KSHV associated diseases have genetic alterations which are driven by chromosomal instability (CIN), as seen in numerous viral-associated cancer cells. Here we examined the molecular mechanism behind KSHV-induced CIN. We showed that the latent antigen LANA, encoded by KSHV, inhibits Bub1 phosphorylation of H2A and Cdc20, and this led to the dislocation of Shugoshin-1. Our studies demonstrated the direct induction of aneuploidy by LANA. The NNLS domain of LANA serves as an anchor for LANA to promote its multiple functions. We also showed that the NNLS polypeptide can antagonize LANA’s inhibition on Bub1 kinase function, and so rescue the aneuploidy induced by LANA. Development of this property of NNLS is potentially useful for targeted elimination of KSHV-associated cancers.

## Introduction

Kaposi’s sarcoma-associated Herpesvirus, also referred as human herpes virus 8 (KSHV/HHV8), a tumor virus, has been documented to be a major contributor to human malignancies and lymphoproliferative disorders, including Kaposi’s sarcoma, Primary effusion lymphoma, and Multicentric Castleman’s disease. KSHV establishes a latent infection in the host after primary infection and continuously expresses genes to facilitate its persistence and evasion from the host immune surveillance [1]. The oncogenic genes, include v-FLIP (ORF71), v-Cyclin (ORF72), LANA (ORF73) and some microRNAs, which manipulate cell cycle regulation, cell growth, proliferation and apoptosis [2]. Disruption of cell cycle regulation by KSHV leads to genome instability and contributes to the oncogenic process [3].

Kaposi’s sarcoma (KS) lesions and KSHV-infected primary effusion lymphoma cells have genome instabilities including chromosomal instability (CIN) [4–6]. There are several checkpoints during cell division important for maintaining genome stability, the G1 checkpoint responsible for G1/S transition, the G2 checkpoint responsible for G2/M transition, and the spindle assembly checkpoint or M checkpoint for the transition from metaphase to anaphase [7]. G1 and G2 checkpoints ensure that there are no errors in DNA replication, and the M checkpoint allows for accurate segregation of the duplicated chromosomes into separated nuclei [8,9]. Alteration of cell cycle progression is a major hallmark of tumorigenesis, and KSHV disrupts the G1 checkpoint through deregulation of the expression levels of p21, p16, p53, E2F-responsive genes, CDK6, Cyclin D, Cyclin E, and Cyclin A [10,11]. KSHV also modulates the ATM/ATR signal pathway associated with the G2/M checkpoint through its interaction with Chk2 [12], resulting in release of the G2/M cell cycle block.

The spindle assembly checkpoint assures genome stability by delaying cell division until chromosomes are correctly assembled and segregated during mitosis [7]. Dysregulation of the spindle assembly checkpoint will lead to aneuploidy [9,13], a form of chromosomal instability that involves frequent cytogenetic changes leading to alterations in chromosome number and aneuploidy, can contribute to the oncogenic process [14]. Bub1, a critical component of the spindle checkpoint complex, plays an important role as a guarantor of chromosomal segregation fidelity [15]. Bub1 defects, more specifically in the Bub1 kinase domain can lead to defects in cell mitosis, aneuploidy and tumorigenesis [16]. Bub1 phosphorylates histone H2A at T120 (H2AT120) [17], and recruits Shugoshin-1 (Sgo1) to kinetochores, protecting cohesin at inner centromeres, kinetochore microtubule stability and tension [18,19]. Loss of Sgo1 is known to lead to the disruption of centromere cohesion and mis-segregation of chromatids [20,21]. Furthermore, Bub1 mediated Cdc20 phosphorylation can inhibit the activities of the APC/C E3 ubiquitin ligase [22–24], and arrest of cell cycle at metaphase until all sister kinetochores are attached to microtubules from opposite spindle poles.

LANA is one of a few proteins expressed during KSHV latent infection. LANA regulates multiple pathways by interacting with host proteins through its amino and carboxy terminal domains [25]. It is also involved in cell cycle regulation with diverse functions. LANA can disrupt cell cycle checkpoint at the G1/S and G2/M transitions through interaction with cell cycle regulators, accelerating cell cycle and cell division [26]. Whether LANA directly interferes with spindle checkpoints is not known. Previously, we showed that LANA induces multinucleation, micronuclei, and mitotic bridges [27,28], and recently showed that LANA degrades Bub1 which contributes to CIN [29]. Here we examined the mechanism through which LANA disrupts Bub1 spindle checkpoint function leading to the displacement of Sgo1. We now demonstrate that LANA promotes mitotic exit, accompanied by abnormal chromosomal segregation and aneuploidy, and blocks phosphorylation of H2A and Cdc20 mediated by Bub1. Inhibition of phosphorylation of H2A at T120 led to the displacement of Sgo1 at the centromeres, and Securin and Cyclin B1 levels were decreased with inhibition of Cdc20 phosphorylation. LANA inhibited the kinase activity of Bub1 through competing interactions with these proteins and interferes with the binding of Bub1 with H2A, or Cdc20. Interestingly, the N terminal fragment of LANA located at 1–87 aa interacted with Bub1, and rescued the effect of LANA’s inhibition of H2A and Cdc20 phosphorylation by Bub1, and thus the induced CIN. This molecular mechanism whereby LANA displaces Sgo1 from the centromere through inhibition of phosphorylation of Cdc20 and H2A by Bub1 now provides a unique understanding of how KSHV induces genome instability and promotes tumorigenesis in KSHV related malignancies.

## Results

### KSHV infection results in mitotic arrest defects of infected cells

**Fig 1:**
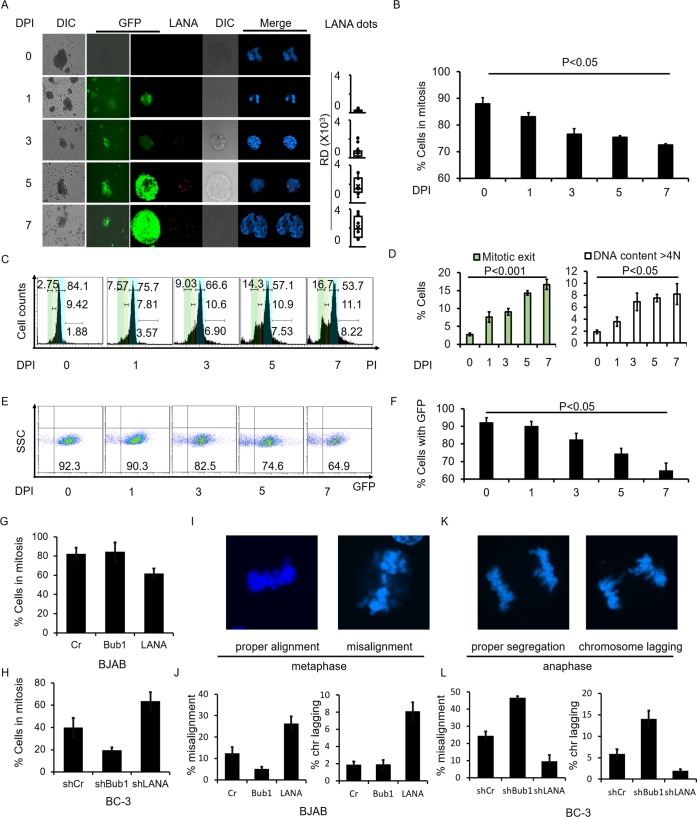
KSHV infection and LANA expression induced mitotic defects and chromosomal instability. The LANA-promoted mitotic defects were observed in KSHV infected BJAB cells. BJAB cells were infected with BAC-KSHV for the indicated days and harvested for immunofluorescence experiment after treatment with Nocodazole for 12 hours. **A,** GFP of KSHV and LANA staining in infected BJAB cells. **B,** Quantitation of cells arrested in mitosis in **A**. **C, D,** DNA content of KSHV infected BJAB cells. Propidium Iodide was used to determine DNA content. Cells with different DNA content were divided into G1, S and G2 phase. The cells which were not in S or G2 phase were regarded as mitotic exit. Green represents G1 phase; Yellow represents S phase; Blue represents G2/M phase. **E, F,** Quantitation of cells with or without GFP in HAC system. BJAB cells were transfected with LANA or Bub1. The effects of mitotic arrest were assessed by the addition of Nocodazole to these cells. **G,** Quantitation of BJAB cells, Bub1 transfected BJAB cells and LANA transfected BJAB cells which were arrested in mitosis in the presence of Nocodazole. **H,** Quantitation of BC-3 shCr, BC-3 shBub1 and BC-3 shLANA cell lines which were arrested in mitosis in the presence of Nocodazole. **I, K,** Representative pictures of chromosome misalignment and chromosome lagging. **J, L,** Chromosome misalignment and chromosome lagging rates in BJAB cells and BC-3 cells.

To investigate whether KSHV infection causes genome abnormalities during cell division in the host cells, we analyzed mitosis in KSHV infected BJAB cells. BJAB cells were infected with BAC-KSHV containing a GFP cassette. After selecting infected cells using 200μg/ml Hygromycin for 3 days, we set the days to Day 1. We then treated the cells with Nocodazole at the indicated time points (Fig 1A). The infected cells were harvested and monitored by immunofluorescence after treatment with Nocodazole for 12 hours. LANA expression levels were monitored up to 7 days post-infection. Its expression was increased as shown by the punctate dots and western blot analyses during the infection (Fig 1A, LANA dots, and S1A Fig). An escape from mitotic arrest under Nocodazole treatment was observed in KSHV infected BJAB cells demonstrating that KSHV can strongly influence bypass of mitotic arrest (Fig 1A and 1B). These cells were arrested in mitosis and those that escaped from mitotic arrest were counted. The results showed a gradual decrease of cells which were arrested in mitosis, which corresponded with an increase in LANA expression levels (Fig 1A and 1B, and S1A Fig). Flow cytometry was also used to determine the cell cycle distribution after BJAB cells were infected with KSHV and treated with Nocodazole for 12 hours. We found that the percentage of cells that escaped from mitotic arrest increased by 5-fold with prolonged days of infection, from 2.75% at day 0 to 16.7% at day 7 (Fig 1C and 1D, green region which represents G1 phase). We also observed an increase in aneuploidy in KSHV infected cells as seen by the results of flow cytometry. The cell percentage with over 4N DNA contents increased by 4-fold from 2% at day 0 to 9% at day 7 (Fig 1C and 1D, DNA over blue region which represents G2/M phase). We further used a novel system for measuring CIN based on the use of a nonessential human artificial chromosome (HAC) to detect the effects of KSHV infection on CIN [30,31]. The HAC carries an EGFP gene, and human fibrosarcoma HT1080 cells with the HAC display green fluorescence, whereas cells losing the HAC do not [30,31]. The cells were infected with KSHV for 7 days and the percentage of EGFP positive cells were monitored during infection. We found that the number of EGFP positive cells decreased with prolonged days of infection, from 92% at day 0 to 65% at day 7 (Fig 1E and 1F). These results demonstrated that KSHV infection disrupts cell mitotic arrest, promoting mitotic escape inducing chromosomal instability.

### LANA encoded by KSHV induces mitotic arrest defects

LANA plays an important role in regulating cell cycle and is involved in aneuploidy according to previous observations from our group [29]. To determine the direct effect of LANA expression on cell mitosis, we monitored mitosis in LANA transfected KSHV negative cells and in LANA deleted KSHV positive cells. KSHV-negative BJAB cells were transfected with LANA or Bub1. Expression of LANA and Bub1 in BJAB and LANA transfected BJAB cells were detected by western blot (S1B Fig). The effects on mitotic arrest were assessed after treatment with Nocodazole. The results showed that in the presence of LANA, 62% of total cells were arrested in mitotic phase (Fig 1G), while 82% and 84% of total cells were in mitotic phase in the control, and Bub1 transfected BJAB cells, respectively (Fig 1G). These results were further corroborated in BC-3 cell lines (Fig 1H). In KSHV LANA positive cells BC-3 control cells and Bub1 knock down BC-3 cells (S1C Fig), there were much fewer cells (40% and 19%, respectively) arrested in the mitotic phase than in LANA knocked down cells (63%) (Fig 1H).

Two known mitotic defects underlying chromosome missegregation [32], chromosome misalignment and chromosome lagging were also examined (Fig 1I and 1K). Chromosome misalignment and chromosome lagging were observed at high rates in LANA transfected BJAB cells (Chromosome misalignment, 26%; chromosome lagging, 8%) and BC-3 cells (Chromosome misalignment, 24%; chromosome lagging, 5.9%) (Fig 1J and 1L). In contrast, chromosome misalignment and chromosome lagging were observed at much lower rates in Bub1 expressed BJAB cells (Chromosome misalignment, 5.1%; chromosome lagging, 1.9%), and LANA knocked down BC-3 cells (Chromosome misalignment, 10%; chromosome lagging, 2%) (Fig 1J and 1L). These results demonstrate that LANA affects chromosome alignment during metaphase which leads to abnormal chromosomal segregation.

### LANA inhibits Bub1 mediated phosphorylation of H2A and Cdc20

**Fig 2:**
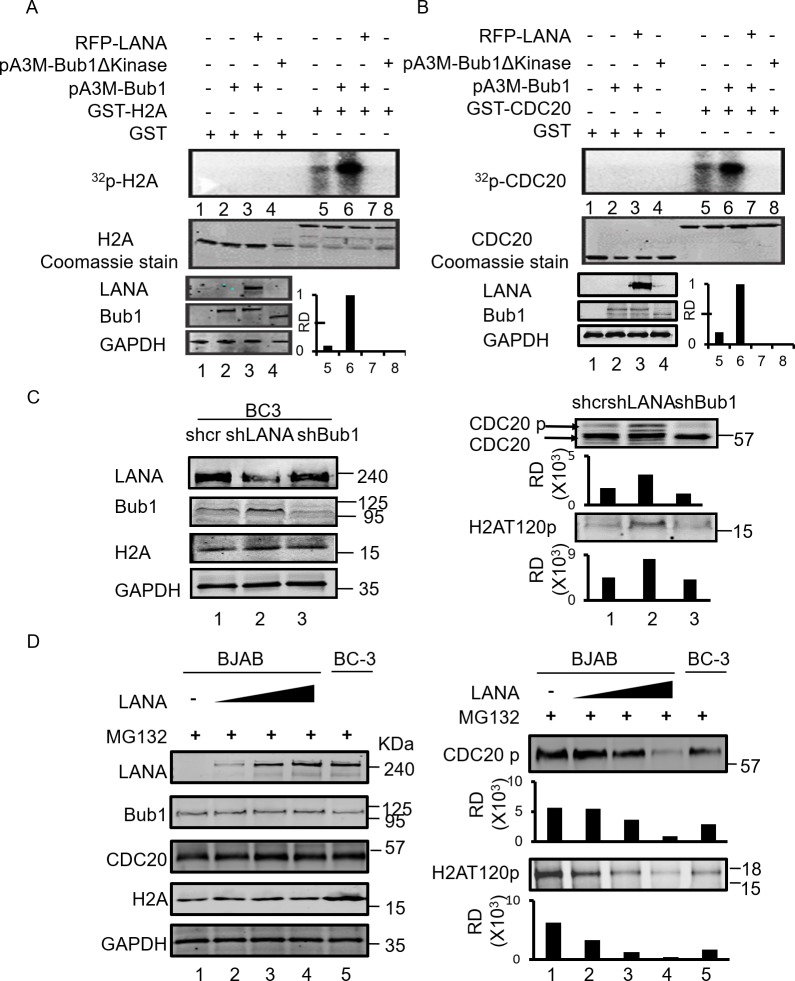
LANA inhibits Bub1-mediated phosphorylation of H2A and Cdc20. **A, B,** To investigate whether the phosphorylation of H2A and Cdc20 by Bub1 were inhibited in the presence of LANA, an *in vitro* kinase assay was performed. HEK293 cells were transfected with Bub1 or co-transfected with Bub1 and LANA. Cells were harvested and immunoprecipitated with anti-myc antibodies 48 hours post transfection. The immune complexes were then incubated with purified GST- tagged H2A or Cdc20 in kinase reaction buffer. **C,** Investigation of the phosphorylation level of H2A and Cdc20 in the absence or presence of LANA by knockdown of LANA in KSHV positive BC-3 cells. **D,** BJAB cells were transfected with different amount of LANA plasmids. 48 hours later, cells were collected and western blot was done with indicated antibodies. BC-3 cells were used as a reference control. RD means relative densities which were quantified using the Odyssey ImageQuant software.

Our previous study showed that LANA contributed to Bub1 degradation which induces chromosomal instability [29]. We now show that LANA positive cells can also exhibit abnormal chromosomal segregation in the presence of Bub1 (S2A Fig). BJAB and BC-3 cells were arrested in metaphase by Nocodazole and treated with MG132 to prevent LANA-mediated Bub1 degradation. The cells which were arrested in metaphase were counted. We found that most of the cells without LANA were arrested in metaphase in the presence of Nocodazole (S2B and S2C Fig). Approximately 40% of the LANA transfected BJAB cells, and BC-3 shCr cells escaped from mitotic arrest even in the presence of Nocodazole and MG132 (S2B and S2C Fig). Recently, it was found that Bub1 phosphorylated histone H2A at residue T120, and recruited Sgo1 to the kinetochores [19]. Sgo1 protects cohesin at the inner centromeres and prevents premature cleavage by Separase [33]. Furthermore, Bub1 mediated Cdc20 phosphorylation is essential for inhibiting the APC/C E3 ubiquitin ligase activity [34]. Phosphorylated H2AT120 and Cdc20 are critical for ensuring that cells maintain chromosomal stability during mitosis. Therefore, we investigated the effect of LANA on Bub1-mediated phosphorylation of H2AT120 and Cdc20. To determine whether phosphorylation of H2A and Cdc20 by Bub1 was dysregulated in the presence of LANA, an *in vitro* kinase assay was performed. HEK293 cells were transfected with Bub1 or co-transfected with myc tagged Bub1 and RFP tagged LANA. Cells were harvested and immunoprecipitated with anti-myc antibodies 48 hours post transfection. The immune complex was incubated with purified GST-tagged H2A or Cdc20. We showed that the phosphorylation levels of H2A or Cdc20 were dramatically increased in the presence of full length Bub1 (Fig 2A and 2B, lane 6) but not kinase domain mutant of Bub1 (Fig 2A and 2B, lane 8). Importantly, once LANA was introduced into the reaction, the phosphorylation of H2A and Cdc20 was substantially reduced (Fig 2A and 2B, lane 7). The phosphorylation level of H2A and Cdc20 in the presence of LANA, or its absence by knocking down LANA in KSHV positive BC-3 cells were further monitored (Fig 2C). We observed that the phosphorylation of H2A and Cdc20 was at low levels in BC-3 shCr cells, which was similar to Bub1 knocked down BC-3 cells. There was an increased level of phosphorylation of H2A and Cdc20 in LANA knocked down BC-3 cells (Fig 2C, lane 1, 3 and 2). We further monitored the phosphorylation of H2A and Cdc20 in the BJAB cells. BJAB cells were transfected with increasing amounts of LANA and 48 hours later, cells were treated with MG132 for 12 hours, collected and analyzed by western blots. The results showed that phosphorylation of H2A and Cdc20 gradually decreased in the presence of an increased concentration of LANA, while the total protein levels of Bub1, H2A or Cdc20 remained unchanged (Fig 2D, right panels). BC-3 cells were used as a reference control. These results demonstrated that LANA can inhibit H2A and Cdc20 phosphorylation.

### Inhibition of H2AT120 phosphorylation affected recruitment of Sgo1 to centromeres and led to loss of cohesion

Since LANA expression inhibited the phosphorylation of H2A at T120 residue, we used immunofluorescence to detect the correlation between LANA and phosphorylated H2AT120 levels. BC-3 shCr, BC-3 shLANA, BJAB and BJAB transfected with LANA were stained with LANA and phosphorylated H2AT120 specific antibodies to determine the levels of signals when coexpressed. When LANA was highly expressed, the phosphorylation of H2AT120 was greatly reduced as indicated by the white arrows (S3 Fig, panels A and C). When little or no expression of LANA was seen, the level of phosphorylated H2AT120 was strongly increased as shown by the red arrows (S3 Fig, panels B and D). We also monitored the localization of LANA, centromere and phosphorylated H2AT120 (S4 Fig). We found that there was a high level of co-localization between H2AT120 and centromeres. However, when LANA was present, the density of H2AT120 was decreased (S4 Fig, compare panels A and D, with B and C). These results demonstrate that LANA inhibits Bub1-mediated phosphorylation of H2A at the T120 residue.

H2AT120 phosphorylation recruits Shugoshin (Sgo1) to kinetochores, and Sgo1 protects cohesins at the inner centromeres [19,35–38]. Furthermore, Bub1 phosphorylation of H2A at T120 prevents premature chromosomal segregation [17]. Therefore, inhibition of H2AT120 phosphorylation will affect recruitment of Sgo1 to centromeres and so promote premature chromosomal segregation. Prolonged loss of H2AT120 phosphorylation will lead to aneuploidy [39].

**Fig 3:**
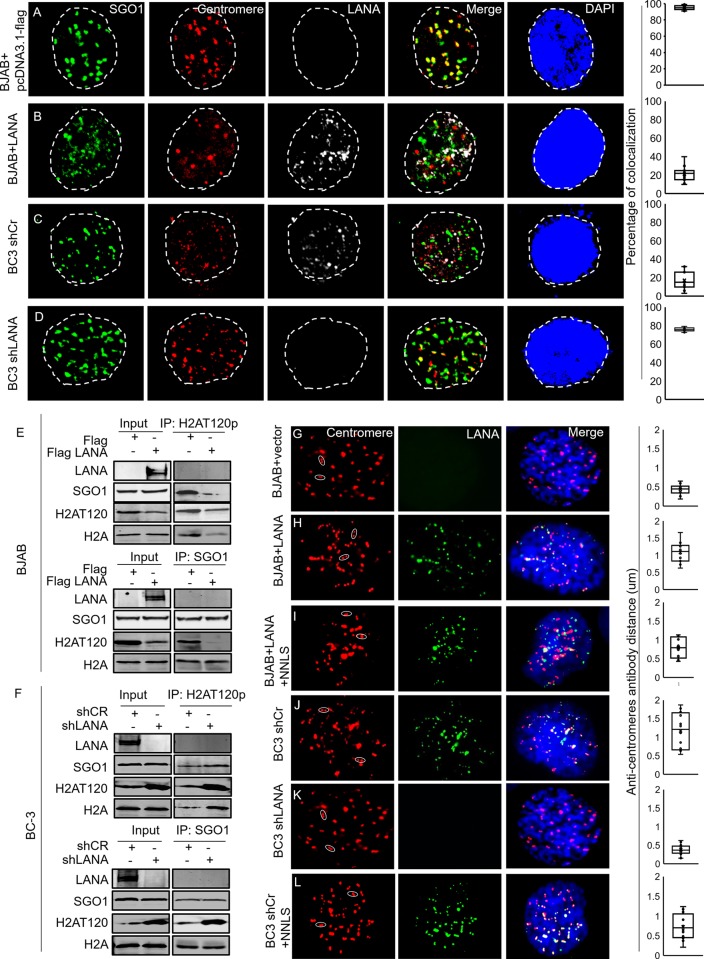
Inhibition of H2AT120 phosphorylation displaces Sgo1 from the centromeres. **A-B,** BJAB cells were transfected with pcDNA3.1-flag-LANA or pcDNA3.1-flag vector. 48 hours later, cells were harvested and fixed for immunofluorescence. **C-D,** BC-3 shCr and LANA knocked down BC-3 cells were harvested and fixed for immunofluorescence. Cells were stained with anti-Sgo1, centromere and LANA antibodies. The columns at right represent colocalization between Sgo1 and Centromere. **E-F,** To detect interaction between phosphorylated H2AT120 and Sgo1, phosphorylated H2AT120 or Sgo1 antibody were used for IP. western blot was done with indicated antibodies. **G-L**, BJAB transfected with empty vector plasmid, BJAB cells transfected with LANA, or LANA plus NNLS; BC-3 shCr, BC-3 shLANA and BC-3 shCr transfected with NNLS were immunostained with anti-centromere antibody (red) and LANA (green). DNA was stained with DAPI (blue). Average distances between paired anti-centromere antibody signals in each cell line are shown in the right panel. The mean scores were examined by using Student’s t-test. The p-value for G-H-I and J-K-L are as following. G-H: p = 5.18731E-06, G-I: p = 0.000881027, H-I: p = 0.021685667, J-K: p = 0.00012466, J-L: p = 0.016699138, K-L:p = 0.00456501.

We further investigated the recruitment of Sgo1 to the centromeres in the presence of LANA (Fig 3). BJAB cells were transfected with LANA expressing plasmids or control plasmids. Cells were fixed and stained with Sgo1, centromere, LANA specific antibodies and DAPI. In the absence of LANA, there was a high level of co-localization between Sgo1 and centromeres (Fig 3A). However, when LANA was expressed, Sgo1 was distributed inside the nucleus and showed minimal (approximately 20%) co-localization with the centromeres (Fig 3B). This was also observed in KSHV positive BC-3 cells positive for LANA, where Sgo1 showed weak co-localization (<20%) with centromeres (Fig 3C). However, there was strong co-localization (>75%) between Sgo1 and centromeres in LANA depleted BC-3 cells (Fig 3D). Importantly, we showed an interaction between phosphorylated H2AT120 and Sgo1. Phosphorylated H2AT120 interacted with Sgo1 in the absence of LANA (Fig 3E and 3F). However, in the presence of LANA, the complex showing the interaction was disrupted. This was largely due to the reduction of H2AT120 phosphorylation (Fig 3E and 3F). We further did reciprocal immunoprecipitation with Sgo1 antibody. The results showed that equal level of total H2A was immunoprecipitated by Sgo1 in the presence and absence of LANA (Fig 3E and 3F), but less H2AT120 was immunoprecipitated. The results demonstrated that the expression level of H2A was not affected by LANA and it is phosphorylated H2AT120 that interacts with Sgo1.

Since Sgo1 protects against the dissociation of cohesion in early mitosis [40], and LANA expression displaces Sgo1, we next monitored the integrity of sister chromatids in the presence of LANA. We found that centromeric signals of chromosomes were separated by a larger distance in LANA expressing cells as indicated by the distance between paired anti-centromere antibody signals (Fig 3G and 3H and 3J and 3K). Interestingly, we found a fragment located at N terminal of LANA (here named NNLS) can rescue the displacement of Sgo1 (S6A Fig and Fig 3I and 3L). We further performed chromosome spreads to observe sister chromatid structure in the absence or presence of LANA. While most of LANA negative BJAB cells or LANA depleted BC-3 KSHV positive cells showed the typical “X-shaped” chromosomes, the sister chromatids were often separated along the whole chromosome length in BC-3 cells and LANA transfected BJAB cells (S5A and S5B Fig). These results demonstrated that inhibition of phosphorylated H2AT120 by LANA severely displaced Sgo1 from centromeres and contributed to premature chromosome segregation during mitosis.

### Inhibition of Cdc20 phosphorylation promoted Cyclin B1 and Securin degradation

**Fig 4:**
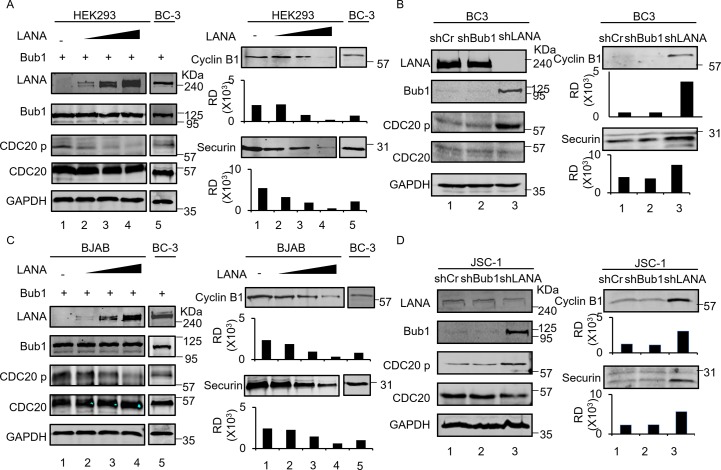
Inhibition of Cdc20 phosphorylation promotes Cyclin B1 and Securin degradation. **A, C,** HEK293 and BJAB cells were transfected with Bub1 and different amount of LANA plasmids (5ug, 10ug and 20ug) or 20ug of empty vectors. 48 hours later, cells were collected and western blot was done with indicated antibodies. BC-3 cells were also transfected with Bub1 and used as a reference control for HEK293 and BJAB. **B, D,** Investigation of the phosphorylation level of H2A and Cdc20 in absence or presence of LANA by knockdown LANA in KSHV positive BC-3 and JSC-1 cells. Cells were collected and western blot was done with indicated antibodies.

Degradation of Cyclin B1 and Securin is crucial for the induced exit of cells from mitosis [41]. APC/C^Cdc20^ promotes metaphase-anaphase transition through Cyclin B1 and Securin degradation via the ubiquitin degradation pathway[42]. Mitotic checkpoint proteins Bub1, Bub3 and MAD family of proteins prevent the E3 ubiquitin ligase APC/C ^Cdc20^ from degrading its targets Cyclin B1 and Securin, arresting cell division at metaphase until all sister kinetochores are attached to microtubules from opposite spindle poles [43,44]. Cdc20 phosphorylation is mediated by Bub1 to inhibit APC/C ^Cdc20^ activity [22,24]. Here we show that Bub1-mediated Cdc20 phosphorylation was decreased in the presence of exogenous Bub1 and LANA expression in HEK293 and BJAB cells (Fig 4A and 4C). Correspondingly, the decrease in Cdc20 phosphorylation resulted in decreased levels of Cyclin B1 as well as Securin (Fig 4A and 4C). The blot for latently-infected BC3 was used as a frame of reference. Based on these results, we see no obvious effects of empty expression vector on the levels of Cyclin B1 and Securin expression compared to LANA. This demonstrated that it was the effect of LANA which mediates the decrease of Cyclin B1 and Securin. We also used two KSHV positive cell lines JSC-1 and BC-3 to determine whether LANA induced inhibition of Cdc20 phosphorylation contributed to decreased Cyclin B1 and Securin protein levels in these pleural effusion lymphoma cells. We found that in the presence of LANA, Bub1 levels were decreased in JSC-1 and BC-3 cells (Fig 4B and 4D). In the absence of Bub1, Cdc20 phosphorylation was inhibited and the protein levels of Cyclin B1 and Securin were dramatically down-regulated (Fig 4B and 4D). LANA knockdown with lentivirus expressed shRNA in these two KSHV positive pleural effusion lymphoma cells resulted in the recovery of the protein levels of Bub1 and up-regulation of Cdc20 phosphorylation (Fig 4B and 4D). Consequently, the protein levels of Cyclin B1 and Securin were rescued (Fig 4B and 4D). These results demonstrated that LANA-mediated inhibition of Cdc20 phosphorylation can lead to down-regulation of Cyclin B1 and Securin and may further promote premature mitotic exit during cell cycle.

### LANA can interact with Bub1 through NNLS domain

**Fig 5:**
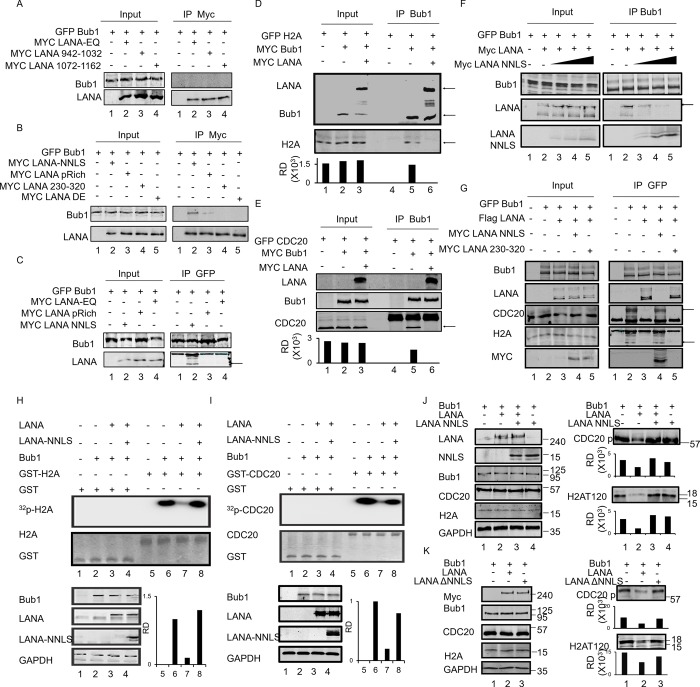
The NNLS domain of LANA interacts with Bub1 and rescues the Bub1 Kinase activity in the presence of LANA. **A, B,** HEK293 were transfected with indicated plasmids. 48 hours later, cells were collected and lysed. 2ug of Myc antibody was used for IP and western blot was done with indicated antibodies. **C,** A reverse CO-IP was performed between Bub1, EQ, pRich, and NNLS domain. GFP antibody was used to do IP and western blot was done with indicated antibodies. **D, E,** HEK293 were transfected with indicated plasmids. 48 hours later, cells were collected and lysed. Bub1 antibody was used to do IP and western blot was done with indicated antibodies. **F,** HEK293 cells were transfected with increasing amounts of NNLS plasmids. 48 hours later, cells were collected and lysed. Bub1 antibody was used to do IP and western blot was done with indicated antibodies. **G,** HEK293 were transfected with indicated plasmids. 48 hours later, cells were collected and lysed. Bub1 antibody was used to do IP and western blot was done with indicated antibodies. **H, I,** HEK293 cells were transfected with indicated plasmids. 48 hours post-transfection, cells were harvested and immunoprecipitated with Bub1 antibodies. The immune complexes were then incubated with purified GST-tagged H2A or Cdc20 in kinase reaction buffer at 30°C for 30 min. the autoradiograph result showed that NNLS can rescue LANA-mediated inhibition of the Bub1 kinase activity (compare lanes 3 and 4, left and right panel respectively). **J,** HEK293 cells were transfected with indicated plasmids. Then western blot was used to validate the in vitro results. **K,** HEK293 cells were transfected with indicated plasmids. 48 hours post transfection, cells were collected and lysed for western blot.

We investigated the mechanism by which LANA inhibited Bub1-mediated H2A and Cdc20 phosphorylation. Our previous research showed that LANA can interact directly with Bub1[29]. To further map the precise interacting domain of LANA with Bub1, a series of LANA truncations were generated and CO-IP experiments with Bub1 and these truncations were performed (S6A Fig and Fig 5A–5C). We showed that none of the truncations derived from the C- terminus of LANA interacted with Bub1 (Fig 5A). The NNLS and pRich domain derived from the N- terminus of LANA can interact with Bub1, although the pRich domain showed a level of interaction that was much reduced (Fig 5B, lanes 2 and 3). To support this result, a reverse CO-IP was performed between Bub1, NNLS, and pRich domains. The results showed that only the domain which contained the NNLS was able to strongly interact with Bub1 (Fig 5C, lane 2).

The inhibition of H2A and Cdc20 phosphorylation may be due to a block in the interaction between Bub1 and its substrates H2A and Cdc20. To support this hypothesis, CO-IP was again performed. As expected, H2A and Cdc20 were found to interact with Bub1 (Fig 5D and Fig 5E, lane 2), while in the presence of LANA, these interactions were substantially diminished (Fig 5D and Fig 5E, lane 3). Since the NNLS is required for the interaction between LANA and Bub1, we wanted to determine if a polypeptide fragment containing the NNLS can compete for interaction between full length LANA and Bub1. We found that the NNLS can competitively disrupt the interaction of LANA and Bub1 (Fig 5F). Further, in the presence of NNLS, the interaction between Bub1 and H2A or Cdc20 was rescued (Fig 5G, lane 2 and 4). These results demonstrated that LANA inhibited Bub1-mediated H2A and Cdc20 phosphorylation through competitive interactions and suggest that the NNLS domain of LANA is an important domain for LANA-mediated inhibition of Bub1 Kinase activity.

### NNLS rescues Bub1 kinase activity in the presence of LANA

Since the NNLS domain can interact with Bub1, this led us to ask whether NNLS facilitated LANA-mediated inhibition of Bub1 Kinase activities. To determine if this hypothesis was correct, *in vitro* kinase assays were performed. HEK 293 cells were transfected with indicated plasmids (Fig 5H and 5I). 48 hours post-transfection, cells were harvested, and lysates were subjected to immunoprecipitation with anti-Bub1 antibodies. The immunocomplex was then incubated with purified GST-tagged H2A or Cdc20 with kinase reaction buffer. Surprisingly, the results showed that the NNLS was unable to inhibit phosphorylated H2AT120 and Cdc20 phosphorylation but rescued LANA-inhibition of Bub1 kinase activity on H2AT120 and Cdc20 phosphorylation (Fig 5H and 5I, lane 3 and 4). Western blot was used to validate the in vitro results and found that the NNLS domain indeed rescued the inhibition of H2AT120 and Cdc20 phosphorylation through LANA while neither NNLS alone or LANA lacking the NNLS (LANA ΔNNLS) had any effects on H2AT120 and Cdc20 phosphorylation (Fig 5J and 5K). These results suggested that LANA can inhibit Bub1’s kinase activity on H2A and Cdc20 through its ability to competitively interact with Bub1. Furthermore, the NNLS domain of LANA is important for regulation of LANA-mediated inhibition of Bub1 kinase activity.

### NNLS can rescue chromosomal instability in the presence of LANA

**Fig 6:**
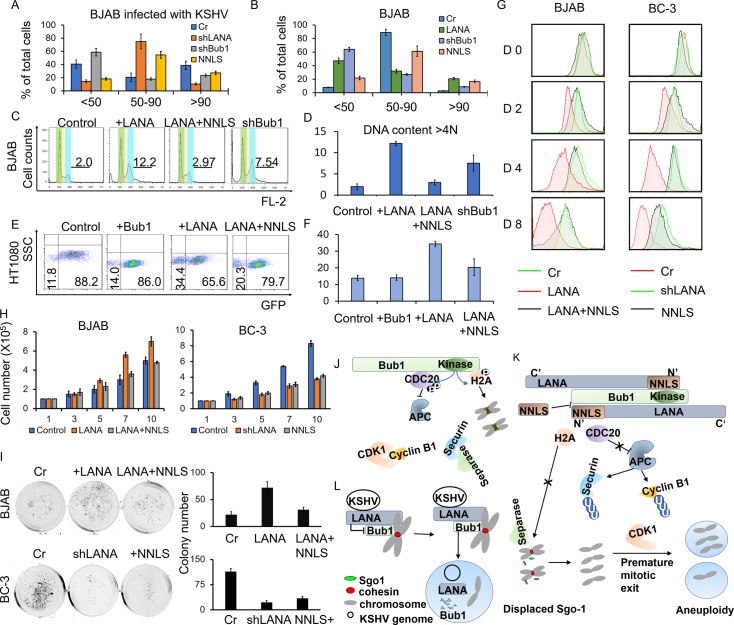
The NNLS domain can regulate LANA mediated chromosomal instabilities and cell proliferation. **A, B,** LANA was knocked down or NNLS was transfected into KSHV infected BJAB cells and empty vector. LANA, LANA plus NNLS (“NNLS”) or shBub1 were transfected into BJAB cells. Cells were treated with Nocodazole for 18h and then fixed with 75% ethanol. As indicated in each panel, Chromosome spread was done to determine the extent of aneuploidy. Chromatids were counted from metaphase spreads. Distribution of chromatids numbers was shown in the indicated cell lines. **C, D,** Flow cytometry was used to detect the DNA content in the indicated cell lines. **E, F,** Quantitation of cells with or without GFP in HAC system in the presence of Bub1, LANA or LANA plus NNLS. Error bars represent standard deviation. Data are representative of 3 independent experiments. Trypan staining, Soft Agar cell growth assay and CSFE staining were used to monitor cell proliferation. We used a pcDNA3.1-Myc vector as the control of LANA-Myc plasmid. BJAB and BC-3 cells were transfected with pcDNA 3.1 Myc empty vector, pcDNA 3.1 Myc-LANA, or pcDNA 3.1 Myc-LANA plus pcDNA 3.1 Myc-NNLS. LANA was knocked down in BC-3 cells with shRNA lentivirus and lentivirus carrying shRNA against luciferase was used as a control in BC-3 cells. **G,** 1X10^5^ cells were stained with 5μM CFSE for 10 minutes at 37°C. Then the cells were washed, cultured, and harvested at indicated time points. Flow cytometry was used to analyze CFSE-labeled cells. **H,** 1X10^5^ cells were plated and cultured for indicated days. Viable cells were counted at the indicated time using trypan staining. **I,** 3X10^5^ cells were plated in soft agar and cultured for 21 days. Cells were stained with Crystal Violet and imaged. **J,** Bub1-mediated Cdc20 phosphorylation inhibits the ubiquitin ligase activity of the APC/C complex. Securin and Cyclin B1 suppress the activity of Separase and CDK1. Bub1-mediated H2A phosphorylation at residue T120 recruits Sgo1 to centromeres. Therefore, cells will be arrested at mitosis until all kinetochores are attached to microtubules. **K,** LANA disrupts the interactions between Bub1, Cdc20 and H2A, inhibiting Cdc20 and H2A phosphorylation. Inhibition of Cdc20 phosphorylation leads to Securin and Cyclin B1 degradation and aberrant activation of APC/C complex. Failure of Sgo1 recruitment because of H2AT120 phosphorylation results in cleavage of cohesin by Separase. This premature mitotic exit induces chromosomal instabilities. LANA interacts with Bub1 through the NNLS domain. The NNLS fragment competitively binds to Bub1 and regulates LANA-mediated inhibition of Cdc20 and H2A phosphorylation. **L.** LANA binds to Bub1 to tether the KSHV viral episomes to host chromosomes. LANA inhibits Bub1’s enzymatic function and further degrades Bub, leading to aneuploidy.

We investigated the role of LANA-mediated Bub1 Kinase inhibition and NNLS rescue effects in the context of aneuploidy. As indicated in each panel (Fig 6A and 6B and S6B and S6C Fig), LANA or Bub1 were knocked down or transfected in the KSHV infected BJAB cells (BJAB-KSHV cells), and LANA or NNLS were transfected into BJAB cells. Chromosome spreads were performed to determine the level of aneuploidy. The chromosome number distribution, which is a marker of chromosomal stability, was also determined after Nocodazole treatment. Representative photographs showing the chromosome spreads were shown in S6 Fig and their quantitation is provided in Fig 6A and 6B. The results showed that the majority of BJAB-KSHV cells had an abnormal number of chromatids (41% had less than 50 chromatids, 39% had more than 90 chromatids), and about 20% exhibited patterns of 50–90 chromatids (Fig 6A). We also showed that the NNLS fragment significantly rescued LANA-induced aneuploidy cells. 55% of NNLS transfected BJAB-KSHV cells had a relatively normal number of 50 to 90 chromosomes (Fig 6A). When LANA was knocked down in BJAB-KSHV cells, an increased number of cells (about 75%) showed a normal number of 50 to 90 chromatids (Fig 6A and S6B Fig). The effect of the rescue function of NNLS was also observed in BJAB cells with LANA or NNLS transfection (Fig 6B and S6C Fig), and so supporting the role of the NNLS in regulating LANA’s activity in suppressing Bub1 Kinase activity.

Flow cytometry was also used to monitor LANA-mediated aneuploidy. BJAB cells were transfected with LANA, LANA plus NNLS, shBub1 or control plasmid. After 48h, the cells were fixed with 75% ethanol overnight and stained with propidium iodide (PI). The DNA content and the distribution of cells at various phases of the cell cycle were analyzed by flow cytometry. The DNA content at G1 phase (Green region), G2/M phase (Blue region) and in cells that contained increased DNA content compared with cells in G2/M (Line at the right of Blue region) was designated as 2N, 4N, and >4N, respectively (Fig 6C). The results showed that a significantly higher percentage of LANA transfected cells (12%) and Bub1 knocked down cells (7.5%) contained >4N DNA content compared to the control (2%) (Fig 6C and 6D). However, aneuploidy rates remained low (3%) if NNLS and LANA were transfected into cells at the same time (Fig 6C and 6D). The HAC system was further used to detect the effects of NNLS on LANA-induced aneuploidy. We monitored EGFP signals using flow cytometry and immunofluorescence microscopy (Fig 6E, 6F and S7 Fig). Co-expression of NNLS with LANA reduced the level of chromosome loss, with about 20% of cells displaying GFP loss (Fig 6E and 6F and S7 Fig). These results demonstrated that NNLS can regulate LANA-mediated aneuploidy.

### NNLS inhibited LANA-mediated cell proliferation

Aneuploidy is closely linked to diseases related to uncontrolled cell proliferation and most tumors exhibit an aneuploidy phenotype [45,46]. Our results above showed that NNLS regulated LANA-mediated inhibition of H2AT120 and Cdc20 phosphorylation. Furthermore, NNLS regulated LANA-induced aneuploidy. LANA can promote cell growth or cell proliferation, and we further asked if NNLS can regulate the effect of LANA on cell proliferation. We used Trypan staining, Soft Agar cell growth assay, and CSFE staining to monitor cell numbers and cell proliferation in BJAB transfected with control plasmid, BJAB transfected with LANA, BJAB transfected with LANA plus NNLS, BC-3, BC-3 transfected NNLS, and LANA knocked down BC-3 cells. The results demonstrated that LANA can dramatically accelerate cell proliferation (Fig 6G, 6H and 6I), while the NNLS dramatically reduced LANA-induced aberrant cell growth both in BJAB cells and BC-3 cells (Fig 6G, 6H and 6I). The expression of NNLS reduced the number of colonies in LANA expressed cells by greater than 50%, and similarly, the cell number was also reduced compared to the control in BJAB and BC-3 cells (Fig 6H and 6I).

## Discussion

The molecular mechanisms which drive virus-associated oncogenic activities are still not completely explored. Here we present a novel mechanism which shows that the oncogenic gene LANA can drive chromosomal instability, a hallmark of KSHV infected cells and KSHV-associated cancer. LANA can inhibit the mitotic checkpoint protein Bub1 kinase activity on H2A and Cdc20, which results in premature chromosomal segregation and mitotic exit. The interaction between LANA and Bub1, H2A or Cdc20, interferes with Bub1-mediated H2A or Cdc20 phosphorylation. The NNLS domain also plays an essential role in the interactions between LANA and Bub1, H2A or Cdc20 as competitive binding of NNLS will rescue LANA’s inhibition of Bub1 kinase activity through its ability to disrupt binding to LANA (Fig 6J and 6K).

The disruption of cell cycle checkpoints by viral factors can provide a favorable environment for the virus to spread or replicate. Genome instability can lead to increased diversity of the genetic context of progeny cells and so contribute to oncogenesis. For example, KSHV entry into host cells can trigger the DNA damage response which will activate ATM/Chk2 and ATR/Chk1 pathways [12,47,48]. This leads to arrest of infected cells at the G1/S checkpoint. LANA releases cells from G1/S arrest through regulation of p16, Rb, p53 and BRD4 [49–52]. LANA also promotes G2/M transition through interaction with Mdm2, p73, ANG and GSK-3 [25]. How KSHV interferes with the mitotic checkpoint is not known. Mitotic checkpoint or spindle assembly checkpoint is a safeguard which prevents mitotic cells from exiting mitosis in the presence of unattached or improperly attached chromosomes, thus avoiding whole-chromosome gains or losses[53]. KSHV related Kaposi Sarcoma has been shown to exhibit chromosomal instability[4,54]. These observations indicate that KSHV infection could predispose cells to malignant transformation through the induction of genomic instability and contributes to the development of KS [4,54]. Previously, our lab has shown that LANA can induce Bub1 degradation and that the process is one factor that leads to aneuploidy in LANA positive cells [29]. Further, we showed that there was abnormal chromosomal segregation in LANA positive cells even when Bub1 was expressed. These results led us to investigate the molecular mechanism of LANA-induced chromosomal instability in greater detail.

The spindle assembly checkpoint complex is a unique machinery composed of Mps1, Bub1, Mad1, Mad2, Bub3, and Bub1-related 1 (BubR1)/Mad3 [55]. Bub1 plays essential roles in spindle assembly checkpoint and is related to recruitment of Aurora B kinase, CENP-E, CENP-F, BubR1, Mad1 and Mad2 [56]. H2A and Cdc20 are the only two characterized substrates of Bub1 [17,21,57]. Histone H2A phosphorylation at T120 residue is vital for Sgo1 localization onto centromeres, which protects cohesin from being cleaved by Separase [19,58]. Previous studies demonstrated that Cdc20 phosphorylation and interaction of Bub1 with Cdc20 is necessary for the function of the spindle assembly checkpoint [59]. Here we provide evidence that in the presence of LANA, H2A phosphorylation at the T120 residue is dramatically reduced and as a consequence, recruitment of Sgo1 to the centromere is disrupted. As expected, we showed that Bub1 phosphorylation of Cdc20 using *in vitro* kinase assays, was substantially reduced in the absence of Bub1 and that Cdc20 phosphorylation was suppressed in KSHV positive BC-3 cells which suggests that Bub1 is essential for Cdc20 phosphorylation. Further, we demonstrated that LANA interaction with Bub1 contributed to the reduction of H2AT120 and Cdc20 phosphorylation with deleterious consequences to chromosomal stability.

The spindle assembly checkpoint is the last opportunity to stop the cell cycle and repair DNA damage in virus infected cells that have escaped the G1 and S phase checkpoints [60]. Spindle assembly checkpoint complex inhibits Cdc20, the co-factor of the E3 ubiquitin ligase APC/C, to delay premature chromosome segregation [55]. This ubiquitin ligase triggers mitotic exit by polyubiquitination of two crucial substrates, Cyclin B1 and Securin [34]. By inhibiting APC/C, the MCC stabilizes these substrates, effectively preventing mitotic exit [53]. Failure of Bub1-mediated inhibition of Cdc20 will lead to premature mitotic exit and eventually chromosomal instability. Oncogenic viruses can manipulate Spindle assembly checkpoint to escape the last checkpoint of cell cycle. Simian virus 40 large T antigen can interact with Bub1 [61]. The interaction between Simian virus 40 large T antigen and Bub1 was shown to compromise the spindle checkpoint and correlate with cell transformation [61]. The HPV E2 protein can also induce chromosomal instability through its binding to Cdc20 and delay Cyclin B1 degradation [62]. This suggests that both compromised and overactivated spindle checkpoint are deleterious to infected cells. During EBV infection it was also observed that the mitotic spindle assembly checkpoint was suppressed which promoted cell survival [63]. Furthermore, previous studies reported that EBNA3C and EBNA2 could abrogate the mitotic spindle checkpoint [64,65]. KSHV infection can also lead to Cyclin B1 degradation [66] and suggests a role for KSHV in dysregulation of the mitotic checkpoint. Here we provided a unique mechanism for degradation of Cyclin B1 and Sgo1 displacement which is directly linked to LANA-mediated inhibition of H2AT120 and Cdc20 phosphorylation, critical for chromosome stability.

The NNLS domain of LANA was previously shown to interact with many host cell proteins [25,67]. This domain may serve as an anchor for LANA to exert multiple functions. Here we show that the NNLS polypeptide can antagonize LANA’s inhibition on Bub1 kinase function, and so rescue the aneuploidy induced by LANA. Polypeptides can be highly selective and efficacious as potential pharmacological agents. Additionally, small molecules which target the NNLS domain can be effective agents for disrupting essential functions of LANA critical for cell-mediated aneuploidy and transformation, which results in the oncogenic phenotype. There is also increased interest in peptides for treatment of a range of cancers in the pharmaceutical industry. Development of this property of NNLS is potentially useful for targeted elimination of KSHV-associated cancers.

## Materials and methods

### Cell lines, plasmids, and antibodies

The plasmids pA3M-LANA, pA3F-LANA, pA3M-Bub1, and GFP-Bub1 have been described previously [29]. Antibodies against MyC (9E10), and LANA were generated from hybridomas. A mouse anti-Flag monoclonal antibody (M2) was purchased from Sigma-Aldrich Corp. (St Louis, MO). Rabbit anti-Bub1 antibodies were purchased from Abcam (Cambridge, MA). The BC-3, JSC-1, BJAB, BC-3 shCr, BC-3 shBub1, BC-3-shLANA, JSC-1 shCr, JSC-1 shLANA and HT1080 HAC cell lines have been described previously [28,50,68,69]. Bub1 or LANA cells were transduced with lentivirus containing specific shRNAs and shRNAs against luciferase were used as a control. The knocked down cell lines were then selected with 1ug/ml puromycin.

### Transfection

10^7^ BJAB cells were transfected by electroporation (220V, 950 μF) with Bio-Rad Gene Pulser II electroporator in 400 ul of serum-free medium. The cells were then transferred to complete RPMI 1640 media, which was preincubated to 37°C. The transfection efficiency of BJAB cells was routinely 30–50% under these conditions.

HEK293 cells were transfected with jetPRIME (Polyplus Transfection, Illkirch, France) according to the instructions.

### IP and Western blotting

Immunoprecipitation (IP) and Western blotting were performed as described previously [70]. Briefly, cells were collected and were lysed in lysis buffer (10 mM Tris, 1% NP-40, 2 mM EDTA, 150 mM NaCl [pH 7.5]) with protease inhibitors. For IP, lysates were incubated with the antibodies indicated in the figures and 30 ul of a 1:1 mixture of protein A/G Sepharose beads at 4°C overnight. The beads were washed with RIPA buffer for 3 times, boiled and were subjected to SDS-PAGE for Western blotting. For the co-IP experiments demonstrating the interaction between LANA and Bub1, cells were treated with MG132 (20 uM) for 12 h before being harvested.

### Detection of chromosomal instability

For the detection of chromosomal instability, cells were harvested and suspended in 1XPBS. The cells were fixed with a fixative (4% PFA containing 0.1% Triton-X100) for 30 min at room temperature (RT) and were then stained with PI for 10min at RT. Then the cells were analyzed using flow cytometry with BD FACSCalibur. For quantitation of the percentage of chromosomal instability, 50,000 cells were collected.

### Metaphase chromosome spread

Nocodazole (0.1 g/ml) was added to the cell culture medium, and cells were allowed to incubate for 12 h before harvesting. The cells were then treated with a hypotonic buffer (0.075 M KCl) for 30 min at RT and were fixed three times, for 10 min each time, in a fixative buffer (methanol: acetic acid, 3:1). After resuspending the cells, the cell suspension was dropped from a distance of about 30 inches onto a slide which was tilted at an angle of about 45° and the suspension allowed to roll across the slide.

### Immunofluorescence

Immunofluorescence was performed as described previously [71]. Briefly, Cells were seeded on glass coverslips in 24-well plates before transfection. After treatment, cells were fixed with 4% paraformaldehyde at 4 °C for 60 min and permeated with 0.2% Triton X-100 in PBS for 10 min. Nuclei were visualized by staining with DAPI for 2 min. Images were acquired using a Fluoview FV300 confocal microscope and FLUOVIEW software was used for image analysis.

### CSFE proliferation assay

Cells were collected and suspended in 1X PBS at a concentration of 1X10^6^ cells/ml. The CFSE solution was added to make a final concentration of 5μM. An equal volume of 1X PBS containing 5% FBS was added after 10 mins incubation at room temperature. Cells were washed three times with 1X PBS containing 5% FBS and equally divided into several plates for incubation. At different time points (0, Day 2, Day 4, Day 8), cells were harvested, washed with ice-cold 1X PBS and resuspended in 5ml 1X PBS, then analyzed on FACScalibur cytometer (Becton-Dickinson Inc., San Jose, CA) and FlowJo software (Treestar, Inc., San Carlos, CA)

### Soft agar assays

The soft agar assays were performed using BJAB and BC-3 cells. Briefly, 1 ml of 0.5% agar in complete RPMI 1640 media was poured into 6-well plates and set aside to solidify for 30 min. 0.5 ml 0.3% agar/medium containing 2×105 cells were added to the previous plates as the middle layer and set aside to solidify for 30 min. Then cells were covered with a top layer of another 1ml 0.5% agar/medium. Colonies were stained with 0.005% crystal violet for 1 hour two weeks later and scanned using a Licor Odyssey system (LiCor Inc., Lincoln, NE). The number of colonies was counted using ImageJ software.

### Statistical analysis

Each experiment was repeated at least three times. The mean scores were examined by using Student’s *t-*test. All statistical tests were performed using Microsoft Office Excel. A *P-*value of 0.05 was considered to indicate a statistically significant difference. A *P-*value of 0.01 was considered to indicate highly statistical significance.

## Supporting information

S1 Fig LANA expression in KSHV infected cells, BJAB and BC-3 cells. **A,** BJAB cells were infected with BAC-KSHV with GFP for the indicated days and harvested for western blot experiment using indicated antibodies. **B,** BJAB cells were transfected with control plasmid, LANA or Bub1. 48-72hs later, cells were harvested for western blot experiment using indicated antibodies. **C,** BC-3 shCr, BC-3 shBub1 and BC-3 shLANA cell lines were collected for western blot experiment using indicated antibodies.
(TIF)

S2 Fig LANA induced abnormal chromosome segregation in the presence of Bub1. **A,** Chromosome misalignment and chromosome lagging. **B,** Bub1 and LANA were transfected into BJAB cells separately. Quantitation of BJAB cells, Bub1 transfected BJAB cells and LANA transfected BJAB cells which were arrested in mitosis in the presence of Nocodazole and MG132. **C,** Quantitation of BC-3 shCr, BC-3 shBub1 and BC-3 shLANA cell lines which were arrested in mitosis in the presence of Nocodazole and MG132.
(TIF)

S3 Fig The phosphorylation of H2AT120 is decreased in LANA positive cells. **A, B,** BC-3 and LANA knocked down BC-3 cells were harvested and fixed for immunofluorescence experiment. Cells were stained with anti- phosphorylated H2AT120, centromere and LANA antibodies. **C, D,** BJAB cells were transfected with LANA. 48 hours later, cells were harvested and fixed for immunofluorescence experiment. Cells were stained with anti-phosphorylated H2AT120, centromere and LANA antibodies. When LANA was highly expressed, the phosphorylation of H2AT120 was low as indicated with white arrows. When there is little or no expression of LANA, H2AT120 was highly phosphorylated as indicated with red arrows.
(TIF)

S4 Fig The localization of phosphorylated H2AT120 and LANA. A, B, BJAB cells were transfected with pcDNA3.1 empty vector or plasmid expressing LANA. 48 hours later, cells were harvested and fixed for immunofluorescence experiment. C, D, BC-3 infected with shCr lentivirus and LANA knocked down BC-3 cells were harvested and fixed for immunofluorescence experiment. Cells were stained with anti-phosphorylated H2AT120, centromere and LANA antibodies. The columns at right represent colocalization between Sgo1 and Centromere.
(TIF)

S5 Fig LANA promoted premature separation of sister chromatids. **A, B,** Chromosome spreads were prepared from mitotic BJAB and BC-3 cells and stained with DAPI.
(TIF)

S6 Fig The NNLS domain can regulate LANA induced aneuploidy. **A,** A series of truncations of LANA protein. **B, C,** LANA was knocked down or NNLS was transfected in KSHV infected BJAB cells and LANA or NNLS were transfected into BJAB cells. BJAB cells and KSHV infected BJAB cells were treated with Nocodazole for 18h and then fixed with 75% ethanol. As indicated in each panel, Chromosome spread was done to determine the extent of aneuploidy.
(TIF)

S7 Fig NNLS regulates LANA induces aneuploidy in the HAC system. Immunofluorescence microscopy detection of HAC system in the presence of Bub1, LANA or LANA plus NNLS. Cells were transfected with pcDNA3.1 empty vector (A), pcDNA3.1 expressing Bub1 (B), LANA (C) or LANA plus NNLS (D). The GFP signals were detected with Immunofluorescence microscopy.
(TIF)
