# Correction: Shugoshin 1 is dislocated by KSHV-encoded LANA inducing aneuploidy

**DOI:** 10.1371/journal.ppat.1007732

**Published:** 2019-04-09

**Authors:** Fengchao Lang, Zhiguo Sun, Yonggang Pei, Rajnish Kumar Singh, Hem Chandra Jha, Erle S. Robertson

Fig 3K contains a duplicate image in which the panel showing the localization of centromere is a duplication of Fig 3G. The correct version of Fig 3 is below.

**Fig 3:**
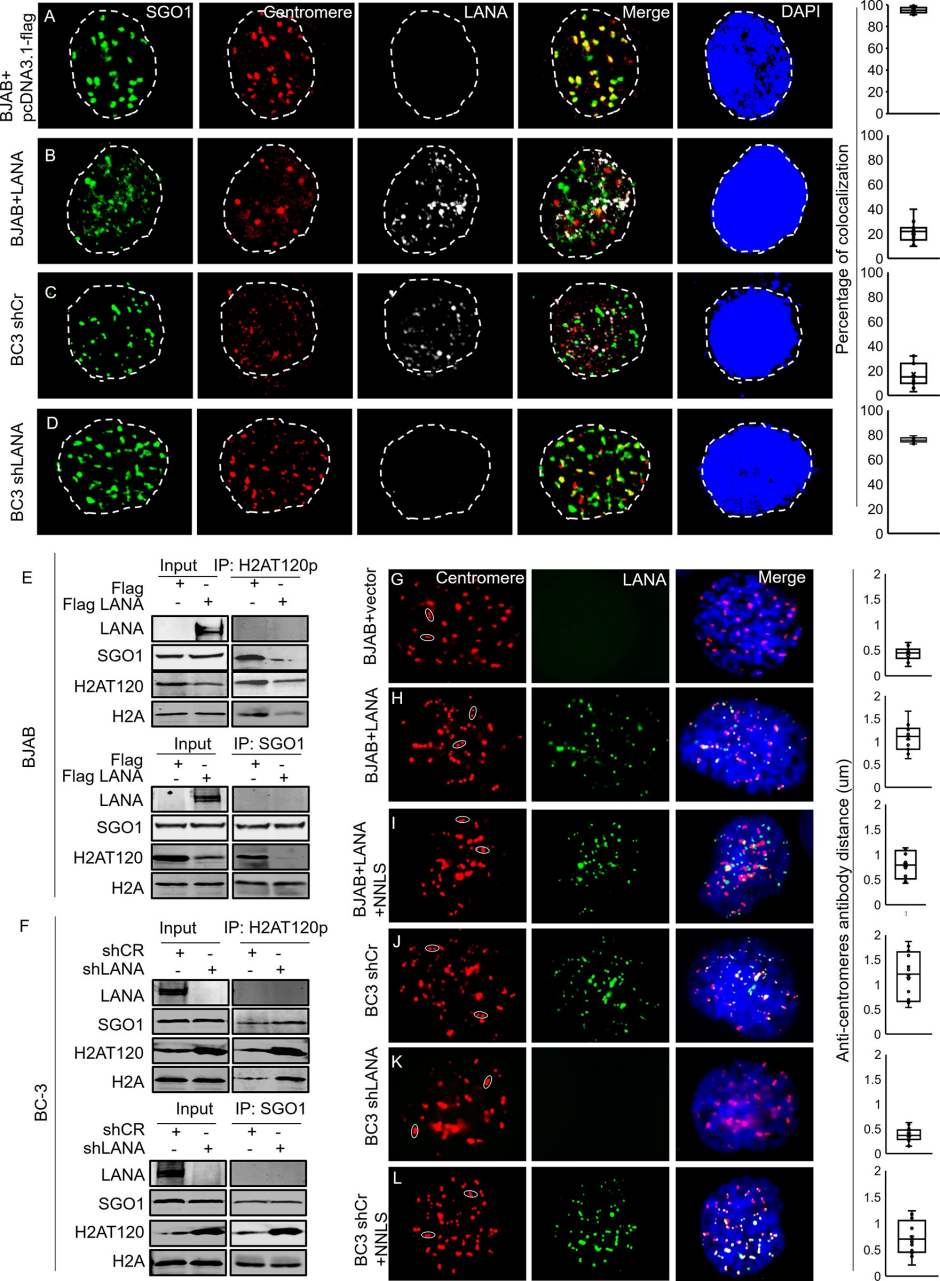
Inhibition of H2AT120 phosphorylation displaces Sgo1 from the centromeres. A-B, BJAB cells were transfected with pcDNA3.1-flag-LANA or pcDNA3.1-flag vector. 48 hours later, cells were harvested and fixed for immunofluorescence. C-D, BC-3 shCr and LANA knocked down BC-3 cells were harvested and fixed for immunofluorescence. Cells were stained with anti-Sgo1, centromere and LANA antibodies. The columns at right represent colocalization between Sgo1 and Centromere. E-F, To detect interaction between phosphorylated H2AT120 and Sgo1, phosphorylated H2AT120 or Sgo1 antibody were used for IP. western blot was done with indicated antibodies. G-L, BJAB transfected with empty vector plasmid, BJAB cells transfected with LANA, or LANA plus NNLS; BC-3 shCr, BC-3 shLANA and BC-3 shCr transfected with NNLS were immunostained with anti-centromere antibody (red) and LANA (green). DNA was stained with DAPI (blue). Average distances between paired anti-centromere antibody signals in each cell line are shown in the right panel. The mean scores were examined by using Student’s t-test. The p-value for G-H-I and J-K-L are as following. G-H: p = 5.18731E-06, G-I: p = 0.000881027, H-I: p = 0.021685667, J-K: p = 0.00012466, J-L: p = 0.016699138, K-L:p = 0.00456501.

## References

[1] Lang F, Sun Z, Pei Y, Singh RK, Jha HC, Robertson ES (2018) Shugoshin 1 is dislocated by KSHV-encoded LANA inducing aneuploidy. PLoS Pathog 14(9): e1007253 10.1371/journal.ppat.1007253 30212568 PMC6136811

