# Supplementary figures and images for: Shugoshin 1 is dislocated by KSHV-encoded LANA inducing aneuploidy
Source: PLoS Pathog. 2018 Sep 13;14(9):e1007253. doi: 10.1371/journal.ppat.1007253 (PMC6136811; doi:10.1371/journal.ppat.1007253)

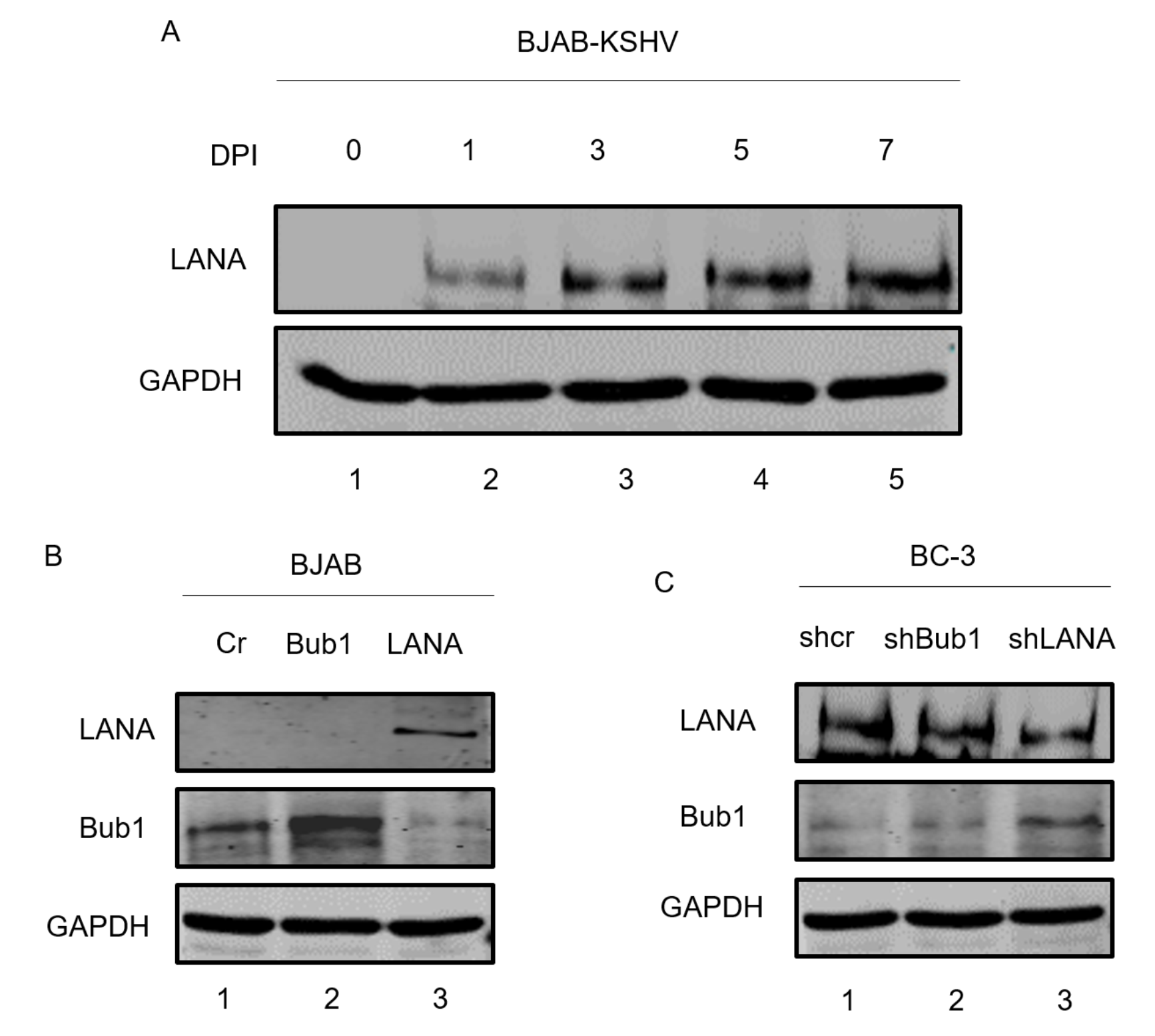

Supplement: S1 Fig — A, BJAB cells were infected with BAC-KSHV with GFP for the indicated days and harvested for western blot experiment using indicated antibodies. B, BJAB cells were transfected with control plasmid, LANA or Bub1. 48-72hs later, cells were harvested for western blot experiment using indicated antibodies. C, BC-3 shCr, BC-3 shBub1 and BC-3 shLANA cell lines were collected for western blot experiment using indicated antibodies. (TIF) [file ppat.1007253.s001.tif]

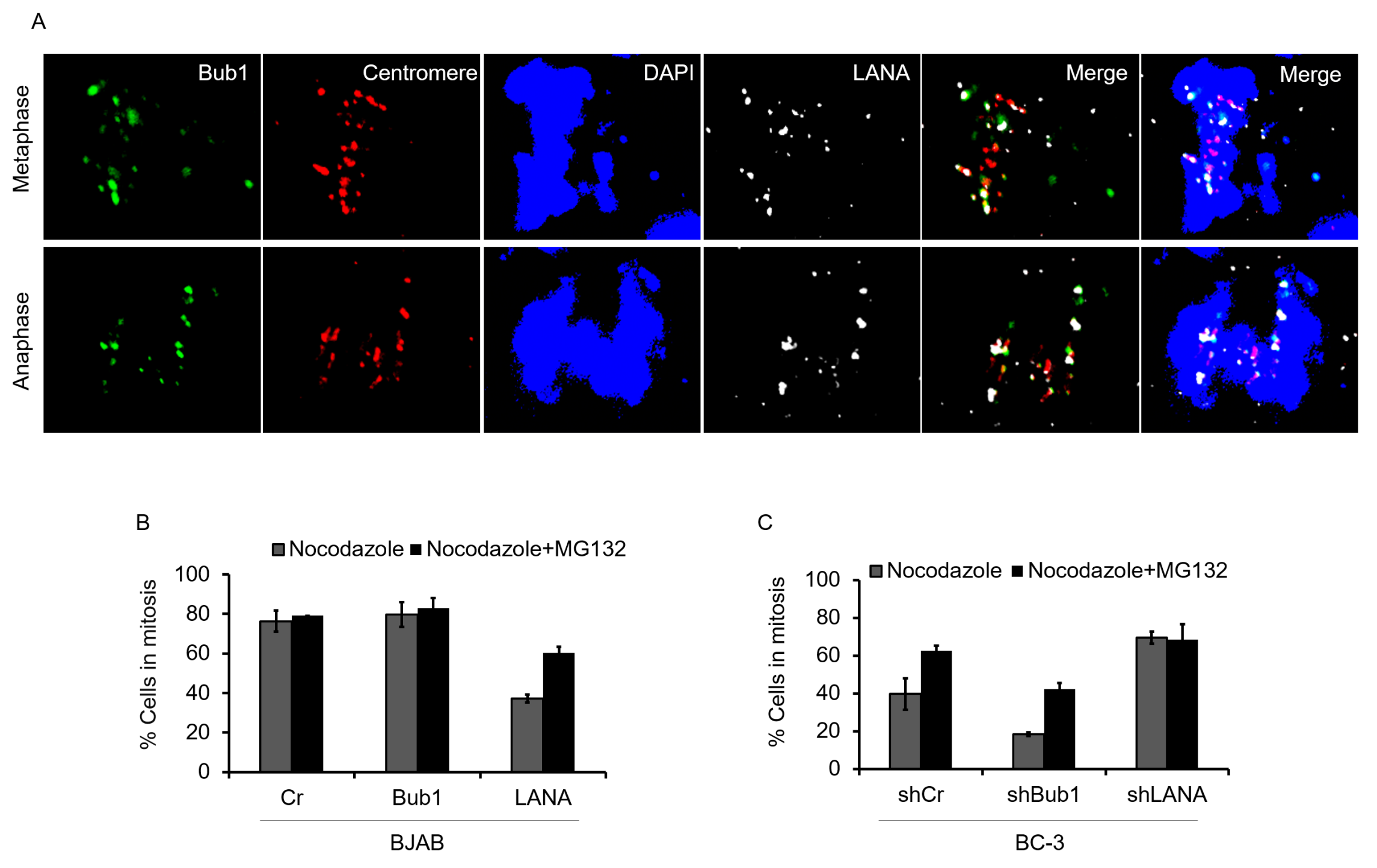

Supplement: S2 Fig — A, Chromosome misalignment and chromosome lagging. B, Bub1 and LANA were transfected into BJAB cells separately. Quantitation of BJAB cells, Bub1 transfected BJAB cells and LANA transfected BJAB cells which were arrested in mitosis in the presence of Nocodazole and MG132. C, Quantitation of BC-3 shCr, BC-3 shBub1 and BC-3 shLANA cell lines which were arrested in mitosis in the presence of Nocodazole and MG132. (TIF) [file ppat.1007253.s002.tif]

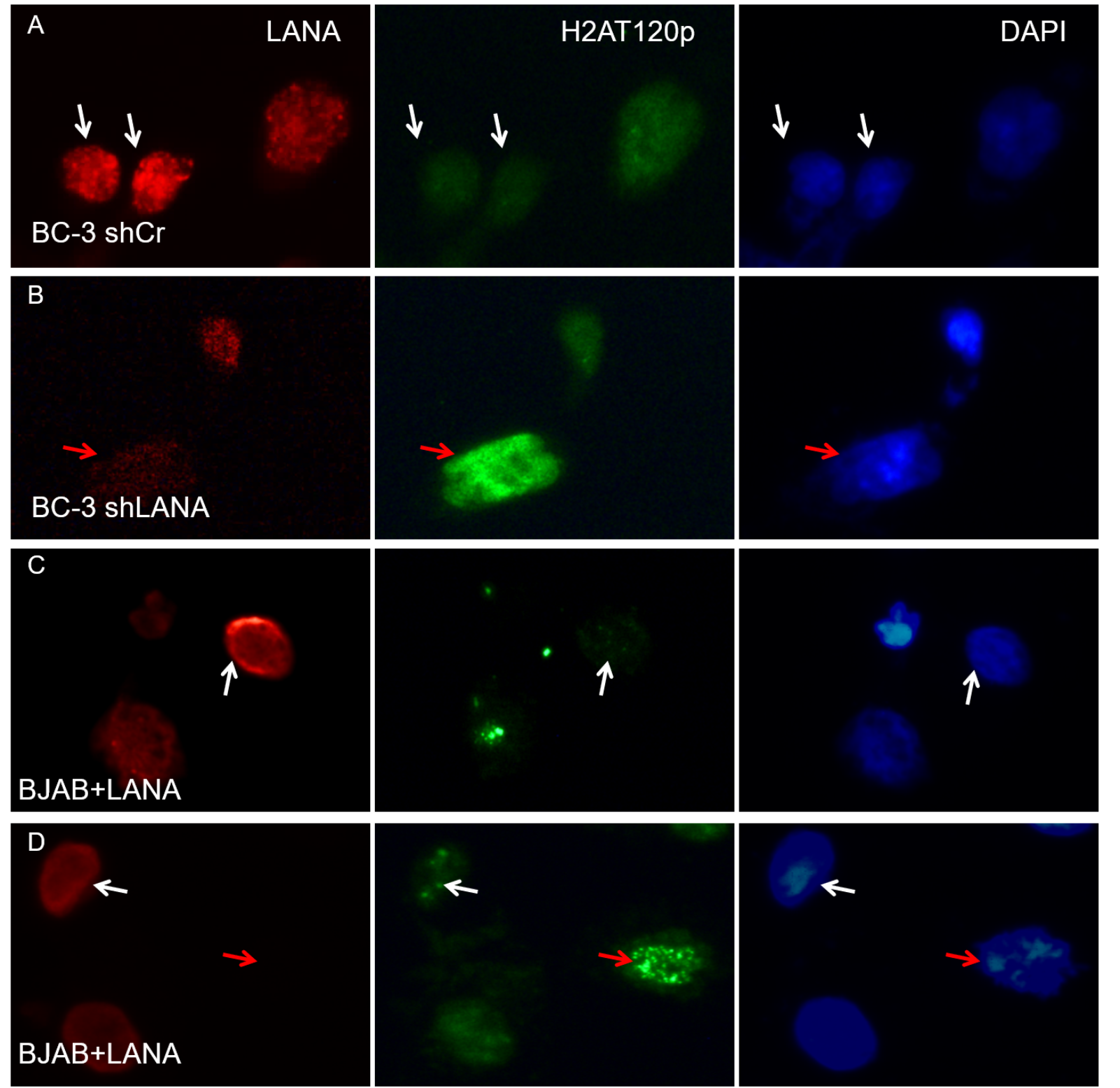

Supplement: S3 Fig — A, B, BC-3 and LANA knocked down BC-3 cells were harvested and fixed for immunofluorescence experiment. Cells were stained with anti- phosphorylated H2AT120, centromere and LANA antibodies. C, D, BJAB cells were transfected with LANA. 48 hours later, cells were harvested and fixed for immunofluorescence experiment. Cells were stained with anti-phosphorylated H2AT120, centromere and LANA antibodies. When LANA was highly expressed, the phosphorylation of H2AT120 was low as indicated with white arrows. When there is little or no expression of LANA, H2AT120 was highly phosphorylated as indicated with red arrows. (TIF) [file ppat.1007253.s003.tif]

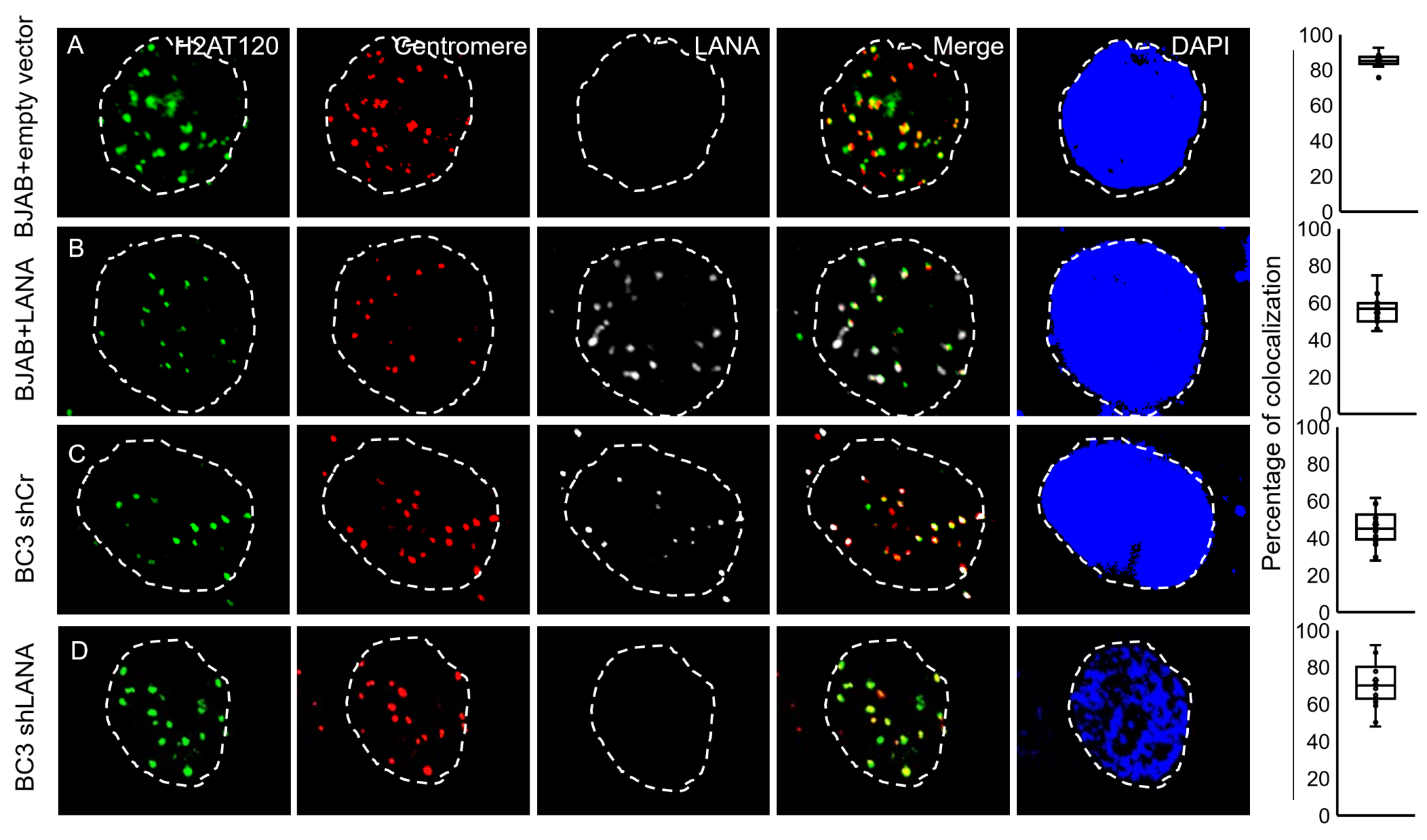

Supplement: S4 Fig — A, B, BJAB cells were transfected with pcDNA3.1 empty vector or plasmid expressing LANA. 48 hours later, cells were harvested and fixed for immunofluorescence experiment. C, D, BC-3 infected with shCr lentivirus and LANA knocked down BC-3 cells were harvested and fixed for immunofluorescence experiment. Cells were stained with anti-phosphorylated H2AT120, centromere and LANA antibodies. The columns at right represent colocalization between Sgo1 and Centromere. (TIF) [file ppat.1007253.s004.tif]

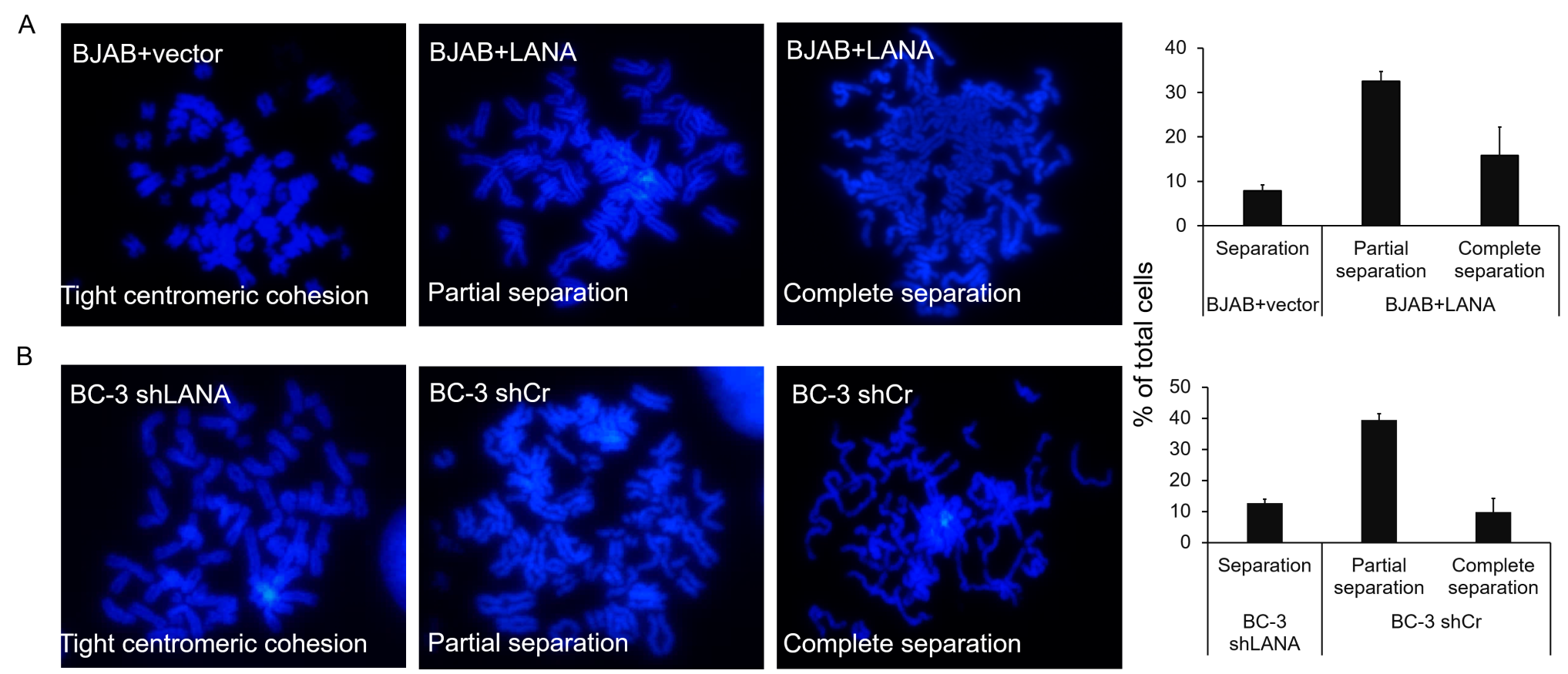

Supplement: S5 Fig — A, B, Chromosome spreads were prepared from mitotic BJAB and BC-3 cells and stained with DAPI. (TIF) [file ppat.1007253.s005.tif]

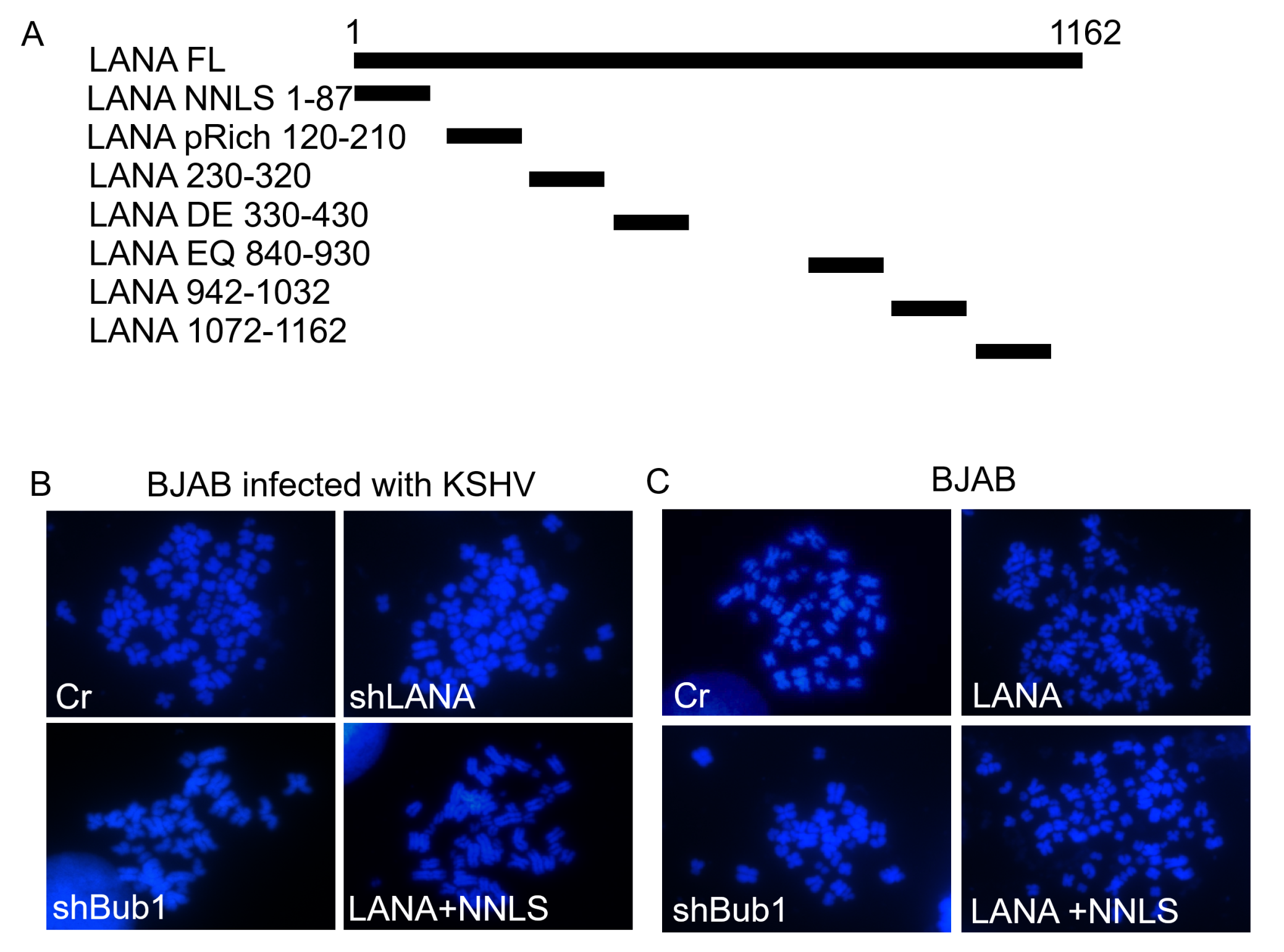

Supplement: S6 Fig — A, A series of truncations of LANA protein. B, C, LANA was knocked down or NNLS was transfected in KSHV infected BJAB cells and LANA or NNLS were transfected into BJAB cells. BJAB cells and KSHV infected BJAB cells were treated with Nocodazole for 18h and then fixed with 75% ethanol. As indicated in each panel, Chromosome spread was done to determine the extent of aneuploidy. (TIF) [file ppat.1007253.s006.tif]

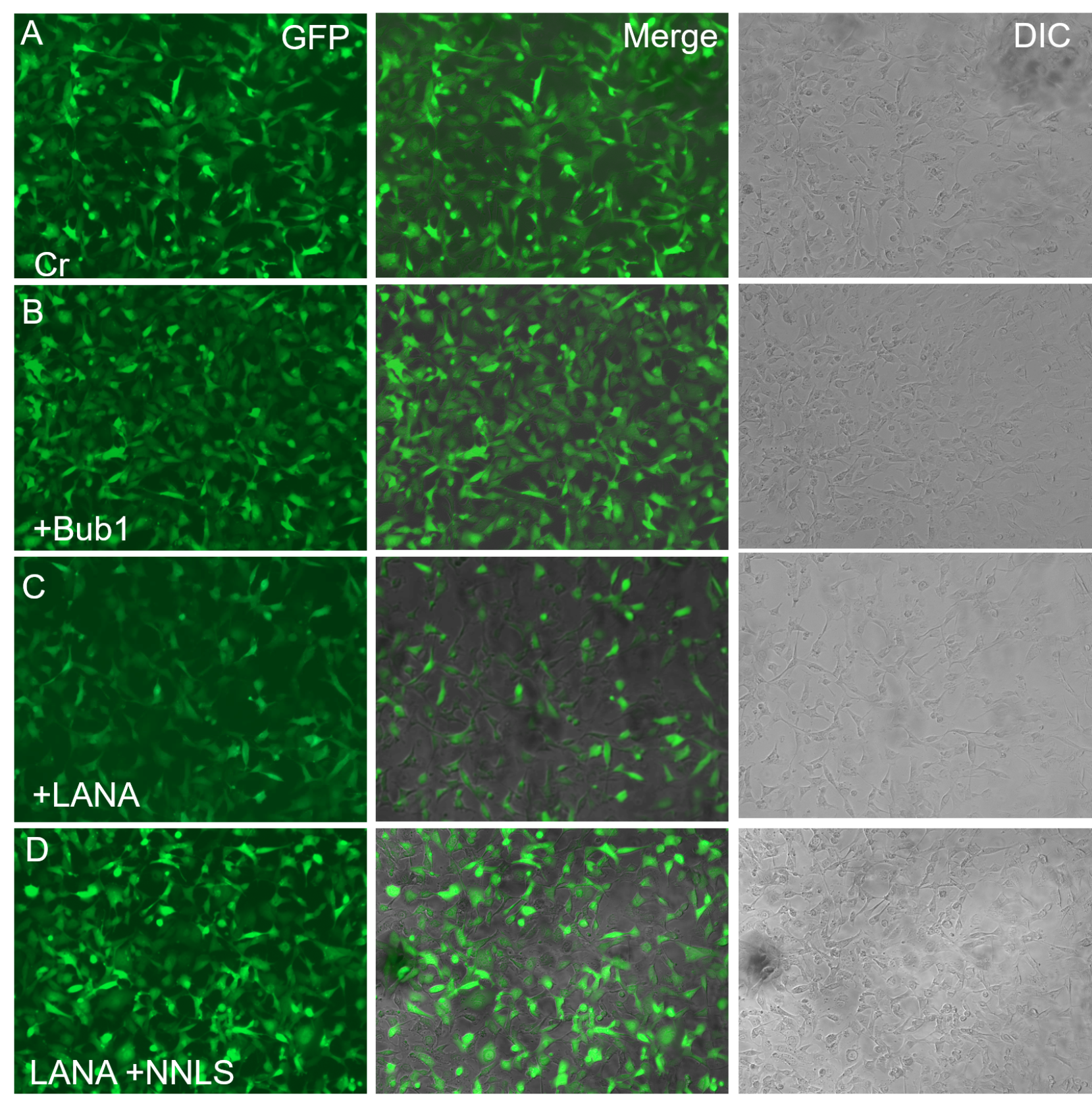

Supplement: S7 Fig — Immunofluorescence microscopy detection of HAC system in the presence of Bub1, LANA or LANA plus NNLS. Cells were transfected with pcDNA3.1 empty vector (A), pcDNA3.1 expressing Bub1 (B), LANA (C) or LANA plus NNLS (D). The GFP signals were detected with Immunofluorescence microscopy. (TIF) [file ppat.1007253.s007.tif]
